# *Jasonia glutinosa* (L.) DC., a Traditional Herbal Tea, Exerts Antioxidant and Neuroprotective Properties in Different *In* *Vitro* and *In Vivo* Systems

**DOI:** 10.3390/biology10050443

**Published:** 2021-05-18

**Authors:** Francisco Les, Marta Sofía Valero, Cristina Moliner, David Weinkove, Víctor López, Carlota Gómez-Rincón

**Affiliations:** 1 Facultad de Ciencias de la Salud, Universidad San Jorge, Villanueva de Gállego, 50830 Zaragoza, Spain; acmoliner@usj.es (C.M.); ilopez@usj.es (V.L.); cgomez@usj.es (C.G.-R.); 2 Instituto Agroalimentario de Aragón, IA2, Universidad de Zaragoza-CITA, 50013 Zaragoza, Spain; msvalero@unizar.es; 3 Departamento de Farmacología Fisiología Medicina Legal y Forense, Universidad de Zaragoza, 50009 Zaragoza, Spain; 4 Instituto de Investigación Sanitaria Aragón (IIS Aragón), 50009 Zaragoza, Spain; 5 Department of Biosciences, Durham University, Durham DH1 3LE, UK; david.weinkove@durham.ac.uk

**Keywords:** medicinal plants, *C. elegans*, acetylcholinesterase, monoamine oxidase A, tyrosinase, herbal medicine, lifespan

## Abstract

**Simple Summary:**

*Jasonia glutinosa* (L.) DC or rock tea (RT) is a plant traditionally used to treat different pathologies. In this study the neuroprotective potential of an ethanolic extract of RT is analyzed. Caenorhabditis elegans model and *in vitro* assays with relevant central nervous system enzymes were used. The results showed antioxidant and neuroprotective potential of this plant.

**Abstract:**

In traditional medicine, *Jasonia glutinosa* (L.) DC or rock tea (RT) has been mainly used to treat digestive and respiratory pathologies but also as an antimicrobial or an antidepressant herbal remedy. An ethanolic extract of RT has been demonstrated to have antioxidant and anti-inflammatory effects, which may be explained by its phytochemical profile, rich in polyphenols and pigments. The aim of this study is to investigate the neuroprotective potential of RT. For this purpose, the ethanolic extract of RT is assayed in *Caenorhabditis elegans (C. elegans)* as an *in vivo* model, and through *in vitro* assays using monoamine oxidase A, tyrosinase and acetylcholinesterase as enzymes. The RT extract reduces juglone-induced oxidative stress in worms and increases the lifespan and prevents paralysis of *C. elegans* CL4176, a model of Alzheimer’s disease; the extract is also able to inhibit enzymes such as acetylcholinesterase, monoamine oxidase A and tyrosinase *in vitro*. Together these results demonstrate that *Jasonia glutinosa* is a good candidate with antioxidant and neuroprotective potential for the development of new products with pharmaceutical interests.

## 1. Introduction

*Jasonia glutinosa* (L.) DC. (Compositae), whose popular name is “té de roca” (rock tea, RT), is a medicinal plant distributed in Mediterranean countries, mainly Spain and France [1]. Ethnobotanical studies on this plant report that its main use, as an infusion, is for the treatment of diarrhea, dyspepsia or abdominal pain [2]. These digestive properties were demonstrated for the first time in a murine model of colitis [3,4]. In these studies, the oral administration of an ethanolic extract of RT (5, 25 and 50 mg/kg) ameliorated colitis symptomatology, prevented the macroscopic damage and histological changes induced by dextran sulfate sodium in mice and normalized the intestinal contractibility and the intestinal total transit disrupted by the colitis [3,4].

In addition to its use as a digestive, *J. glutinosa* has been widely used for the treatment of respiratory or infective pathologies, hypertension, pain, emesis or even mood disorders [2,5,6,7]. Different studies reported an ethnopharmacological use of RT as a “stimulant”, capable of improving mood and clearing the mind [6,7].

Some of these effects could be explained by anti-inflammatory [4,8] or antioxidant effects [4,5,9], which are related to its phytochemical composition. A recent *in vivo* study has shown that dietary supplementation with RT (10 or 30%) for 15 or 30 days in sea bream (*Sparus aurata L.*) improves short-term immunostimulatory capacity (15 days) and maintains antioxidant capacity in the long-term (30 days) [10].

**Figure 1:**
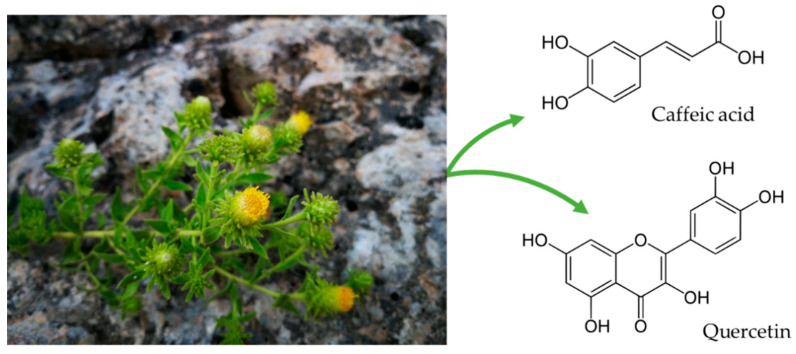
*J. glutinosa* photography taken by authors and the main phenolic compounds of the extract.

Different studies have shown that *J. glutinosa* is rich in polyphenols, terpenes, esters, alkanes, lactones or flavonol glucopyranoside [1,4,9]. High-performance liquid chromatography with a diode-array detector (HPCL-DAD) analysis of the ethanolic extract of RT showed a rich content of phenolic compounds (134.4 mg/g, dry extract) and pigments (0.27 mg/g, dry extract) (Figure 1). Among the 15 phenolic compounds detected, 10 were phenolic acids and 5 flavones. 3,4-di-O-caffeoylquinic acid, 3,5-di-O-caffeoylquinic acid, 4,5-di-O-caffeoylquinic acid, 1,5-di-O-caffeoylquinic acid were the phenolic acids most represented (70% of the total phenolic content). The most abundant flavonoid is quercetin-3-O-galactoside (50% of total flavonoid). In respect to pigments, two carotenoids, chlorophylls and xanthophylls were detected. Lutein represented 55% of the total pigments [4].

Oxidative stress and inflammation have been linked to the aging process as triggers of various diseases, including neurodegeneration. *J. glutinosa*, with a phytochemical composition rich in polyphenols, has previously demonstrated antioxidant and anti-inflammatory effects, and its use as an antidepressant has also been reported by certain inhabitants of Spain. However, there are no scientific reports showing the potential of RT as a neuro-therapeutic agent. Therefore, the objective of this work is to study the antioxidant and neuroprotective activity of RT extract in a *Caenorhabditis elegans (C. elegans)* model, studying the effect of *J. glutinosa* on stress resistance, lifespan and amyloid toxicity and *in vitro* analyzing its ability to inhibit the enzymes of the nervous system such as acetylcholinesterase (AChE), monoamine oxidase A (MAO-A) and tyrosinase (TYR), involved in neurotransmitters’ metabolism. These enzymes participate in the elimination of biogenic amines such as acetylcholine, serotonin and catecholamines (dopamine, epinephrine, norepinephrine), which are involved in the development of different pathologies of the nervous system such as Alzheimer’s, Huntington’s and Parkinson’s. Therefore, the inhibitory substances of these enzymes could help the treatment of these diseases.

## 2. Materials and Methods

### 2.1. Reagents and Chemicals

The following chemical reagents: gallic acid, xanthine, NBT (nitroblue tetrazolium), xanthine oxidase, DPPH (2,2 Diphenyl 1 picrylhydrazyl), galantamine, ATCI (acetylthiocholine iodide), DTNB (5,5′-dithiobis-(2-nitrobenzoic acid)), Tris, vanillic acid, 4-aminoantipyrine, horseradish peroxidase, acetylcholinesterase, tyramine, MAO-A, L-DOPA (levodopa) and tyrosinase were obtained through Sigma-Aldrich (Madrid, Spain). Clorgyline and α-Kojic acid were sourced from Cymit quimica (Barcelona, Spain). Na_2_CO_3_, HCl, NaCl, Methanol and potassium phosphate were acquired from Panreac (Barcelona, Spain). Juglone (5-hydroxy-1,4-naphthoquinone) and FUdR (5-fluoro-2′-deoxyuridine) were sourced from Alfa Aesar (Ward Hill, MA, USA). *C. elegans* strains and Escherichia coli OP50 were obtained from the Caenorhabditis Genetics Center (CGC, Minneapolis, MN, USA).

### 2.2. Plant Material and Extraction

*Jasonia glutinosa* ethanolic extract was obtained as described in Valero et al., (2015) [11] and a plant voucher was kept in the Universidad San Jorge (ref. 001-2012). The ethanolic extract was analyzed phytochemically in a previous author’s study using HPLC-DAD, determining its composition in phenolic compounds and pigments [4].

### 2.3. Caenorhabditis elegans Studies

#### 2.3.1. *C. elegans* Strains and Maintenance Conditions

This study used the wild-type strain of *C. elegans* (N2) and a transgenic strain (CL4176 (smg-1 ts 131 (myo-3/Aβ1–42 long 3′-UTR)). The *C. elegans* CL4176 strain contains a temperature-sensitive transgene that expresses the human amyloid peptide β1–42 in muscle, which causes paralysis in worms.

The strains were maintained at 16 °C (CL4176) or at 20 °C (N2) on nematode growth media (NGM) agar plates seeded with *Escherichia coli* OP50 as a food source. Synchronized worms were obtained using an alkali-bleaching method [12] for the N2 strain and egg-laying for the CL4176 strains.

#### 2.3.2. Assessment of Resistance to Lethal Oxidative Stress

Synchronized L1 worms were cultivated in NGM agar plates in the presence of different concentrations of RT extract (5, 10, 20 and 50 µg/mL) or in its absence (control). Under these conditions, the worms were incubated at 20 °C until the first day of adulthood. RT-treated and control adult worms were washed with sterile water and transferred to 96-well microplate with NGM agar containing 150 µM juglone (5-hydroxy-1,4-naphthoquinone), which produces lethal oxidative stress. After an incubation period of 24 h at 20 °C, survival was evaluated by the response to a mechanical stimulus [13]. The number of alive and dead worms was recorded and the survival rate % (% SR) was calculated:

% SR = (Nº of worms alive x 100)/Total number of worms(1)

Each concentration was tested in triplicate and 100 nematodes were used per assay.

#### 2.3.3. Lifespan Analysis

The lifespan of the wild-type *C. elegans* (N2) was tested using different concentrations of RT (5, 10, 20, 50 and 100 µg/mL) and compared with untreated controls following the method of Solis and Petrascheck [14]. Synchronized wild-type L1 larvae were transferred to 96-well plates (7–18 worms/well) and were cultured in an S-complete medium containing *E. coli* OP50 (1.2 × 10^9^ bacteria/mL). On the first day of adulthood, FUdR (0.06 mM) was added to sterilize the adults (day 0). RT extracts were added 24 h later. Survival of nematodes was scored every two or three days. The scoring method was the same as used for the juglone oxidative stressed assays. Results are expressed as survival rate % (Equation (1)) and mean lifespan. Each assay was repeated three times and used 150 nematodes per condition.

#### 2.3.4. Paralysis Assay

*In vivo* neuroprotective effects of RT extract were evaluated using the strain CL4176 according to previous research protocols [15]. The strain CL4176 was egg-synchronized onto the NGM plates seeded with *E. coli* containing 0, 5, 10 and 25 μg/mL of the RT extract and cultured at 16 °C. The temperature was changed to 25 °C to induce expression of the amyloid-β (Aβ) transgene 38 h later (day 0). Paralysis was scored twice a day for five days [16]. Worms that exhibited pharyngeal pumping, but did not move, or only moved the head after being touched with a platinum wire, were scored as paralyzed. The test was performed with 100 nematodes per condition.

### 2.4. Bioassays Regarding CNS Enzymes

#### 2.4.1. Acetylcholinesterase Inhibition

Ellman’s method was performed in 96-well microplates using a microplate reader to measure absorbance as previously described [17]. Each assay well contained a mixture of 25 µL 15 mM ATCI in Milipore water, 125 µL 3 mM DTNB in buffer C (50 mM Tris-HCl, pH = 8, 0.1 M NaCl, 0.02 M MgCl_2_ 6H_2_O), 50 µL buffer B (50 mM Tris-HCl, pH = 8, 0.1% bovine serum) and 25 µL RT extract at different concentrations (20.00, 10.00, 5.00, 2.50, 1.25 and 0.63 µg/mL) in buffer A (50 mM Tris-HCl, pH = 8). At last, 25 µL AChE enzyme (0.22 U/L) was added to begin the reaction. Controls and blanks were performed, containing buffer A instead of samples and a buffer instead of the enzyme, respectively. Absorbance was read 13 times every 13 s at 405 nm. Galantamine was used as a reference inhibitor.

#### 2.4.2. Monoamine Oxidase A Inhibition

The MAO-A activity was performed in a 96-well microplate using a technique previously described [18]. The assay mixture contained 50 µL RT extract at different concentrations (0.0001, 0.0010, 0.0100, 0.1000, 1.0000 and 10.0000 mg/mL), 50 µL chromogenic solution (0.8 mM vanillic acid, 417 mM 4-aminoantipyrine and 4 U/mL horseradish peroxidase in potassium phosphate buffer pH = 7.6.), 100 µL 3 mM tyramine and 50 µL 8 U/mL MAO-A. Controls and blanks were also performed, with solvent instead of samples and a buffer instead of the enzyme, respectively. The absorbance was read at 490 nm every 5 min for 30 min. Clorgyline was used as a reference inhibitor.

#### 2.4.3. Tyrosinase Inhibition

The TYR activity was assessed in 96-well microplates using a procedure previously described [19]. The reaction mixture contained 10 µL RT extract at different concentrations (0.001, 0.010, 0.100, 0.500, 1.000, 2.500 and 5.000 mg/mL), 40 µL of L-DOPA, 80 µL phosphate buffer, pH = 6.8 and 40 µL tyrosinase; these were mixed in each well. Controls and blanks were also performed with 50 µL solvent instead of samples and 50 µL buffer instead of the enzyme, respectively. Absorbance was read at 475 nm. α-Kojic acid was used as a reference inhibitor.

### 2.5. Statistical Analysis

Data were expressed as mean ± SEM and for statistical analysis, GraphPad Prism version 6.0c (GraphPad Software, San Diego, CA, USA) was used. Extract concentration was required to inhibit 50% of the activity of the nervous system enzymes, IC_50_; this was estimated using non-linear regression. ANOVA following by Tukey’s multiple comparisons test were used to evaluate resistance to oxidative stress. Lifespan and paralysis curves were tested using log-rank for significant fit to Kaplan–Meier survival curves. The significance level was set to *p* < 0.05.

## 3. Results

### 3.1. Rock Tea Extract Improved the Stress Resistance of C. elegans

**Figure 2:**
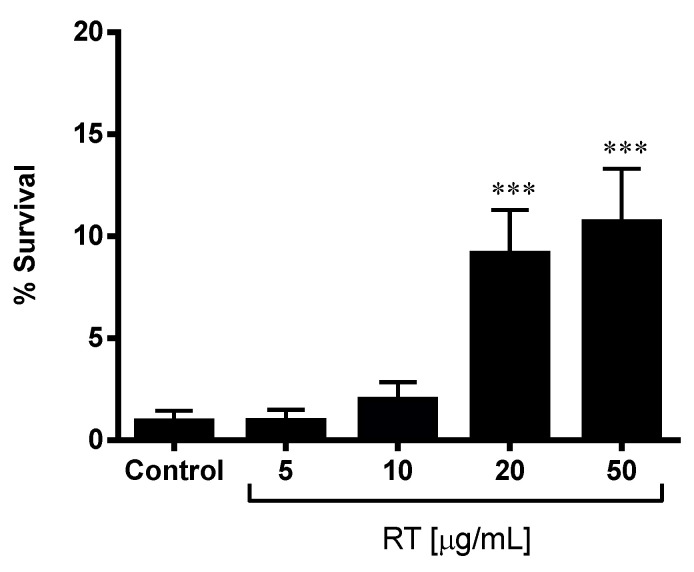
Rock tea (RT) extract increases the survival of wild-type *C. elegans* exposed to lethal oxidative stress. L1 worms were incubated on treatment plates with different doses of RT (5, 10, 20 and 50 µg/mL) until they reached adulthood and then exposed to juglone (150 µM) for 24 h. The results represent the mean ± SEM of the values from three independent experiments. The significance for the differences between the control and pre-treated worms is *** *p* < 0.001.

To evaluate the antioxidant effect of *J. glutinosa*, wild-type *C. elegans* were pre-treated with RT extract (5, 10, 20 and 50 µg/mL) for 24 h and then exposed to a lethal dose of juglone, a natural pro-oxidant. As Figure 2 shows, this treatment resulted in 0.92% ± 0.52 survival of the control group. Pre-treatment with RT extract improved the survival rate of the worms in a dose-dependent manner. Groups pre-treated with the highest doses of RT extract, 20 and 50 µg/mL, significantly increased the survival rate compared to the control group, with rates of 9.15% ± 2.13 (*p* < 0.001) and 10.69% ± 2.51 (*p* < 0.0001), respectively. These results indicate that the RT extract improves oxidative stress resistance in *C. elegans*, protecting them from oxidative stress.

### 3.2. Rock Tea Extract Increased C. elegans Lifespan

**Figure 3:**
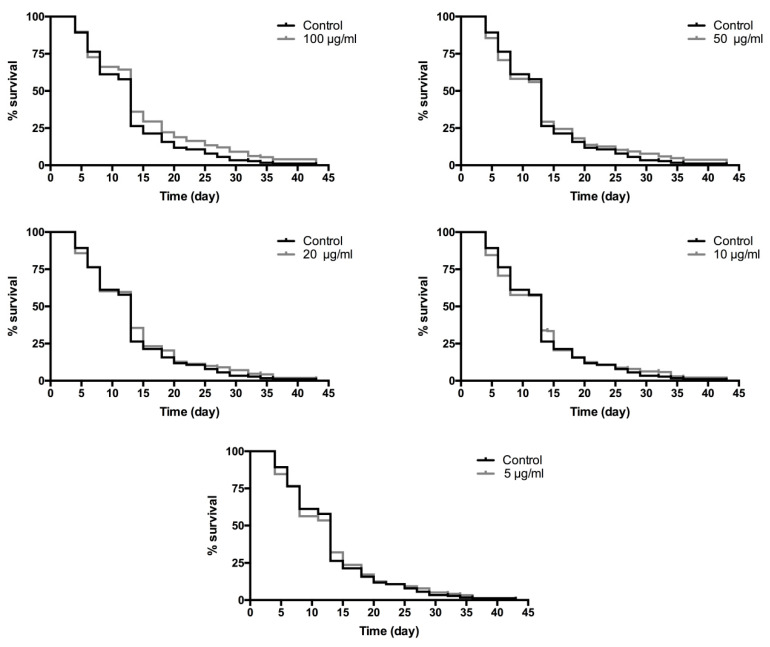
Lifespan curves of wild-type *Caenorhabditis elegans (C. elegans)* in a liquid medium supplemented with different concentrations of RT extract (0–100 µg/mL) at 20 °C. Synchronized worms were exposed to the extract from the second day of adulthood (day one). Scoring of survival was carried out three times per week until all worms died. The results are representative of three independent biological replicates. The curves were analyzed using a long-rank test. Differences in the survival curves between the treatment and control groups were found at the dose of 100 µg/mL, with a *p*-value of 0.0153.

With the object to study the effect of RT extract on lifespan, wild-type *C. elegans* grown at 20 °C in a liquid medium containing different doses of RT (5, 10, 20, 50 and 100 µg/mL) were used. *C. elegans* is a model widely used to study aging and age-related disorders due to the good conservation of the biochemical pathways and their short life cycle [20]. The lifespan curves showed that RT extract increases the lifespan of the worms in a dose-dependent manner (Figure 3). Significant differences were found (*p* < 0.05) in survival curves between the control group and the worms treated with 100 µg/mL of RT extract (12.8 ± 0.56 days vs. 14.7 ± 0.58 days, respectively). The maximum lifespan, understood as the average lifespan of 10% of each population living longer, increased by 11.36% at doses of 20 and 50 µg/mL of RT and 13.81% at the dose of 100 µg/mL of RT with respect to the control. These results show that RT extract presents a positive effect on *C. elegans* lifespan.

The means of lifespan were 12.8 ± 0.56 days in the control and 13 ± 0.57, 13 ± 0.56, 13.45 ± 0.58, 13.42 ± 0.57 and 14.7 ± 0.55 days in the RT 5, 10, 20, 50 and 100 µg/mL groups, respectively. The results of lifespan experiments were analyzed using the Kaplan-Meier survival model and for significance by means of a log-rank pairwise comparison test between the control and treatment groups. Differences in survival curves between the treatment and control groups were found for 100 µg/mL with a *p*-value of 0.0153.

### 3.3. Rock Tea Extract Delays the Onset of Paralysis Induced by Aβ Peptide

**Figure 4:**
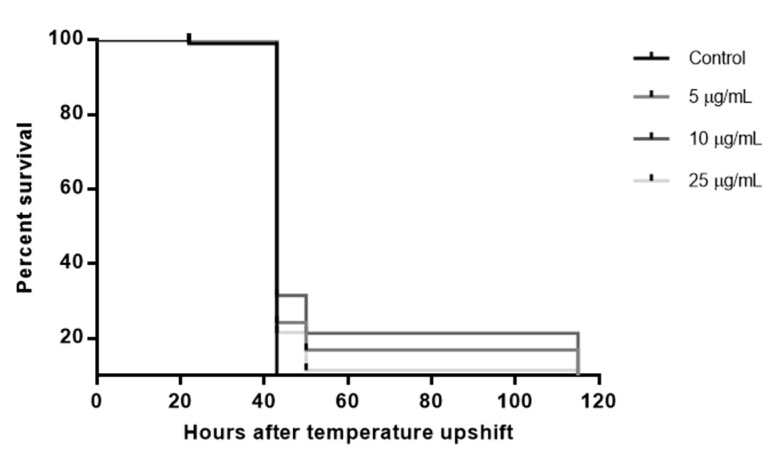
Effect of RT extracts on Aβ-induced paralysis in transgenic *C. elegans* CL4176. The statistical significance of the difference between the curves was analyzed using a log-rank (Kaplan–Meier) statistical test, which compares the survival distributions between the control and treatment groups. Differences in the survival curves between the treatment and control groups were found (*p* < 0.0001).

Although there are multiple factors involved in Alzheimer’s disease, numerous studies have shown that neuronal accumulation of the Aβ peptide plays a central role in the development of the disease [21]. To further investigate the *in vivo* neuroprotective effect of rock tea, an examination was carried out as to whether diet supplementation may affect the progression of paralysis induced by Aβ toxicity in the *C. elegans* transgenic strain CL4176. This strain has proven to be a good model for screening potential neuroprotective natural products [15,22,23]. Nematodes were exposed to different concentrations of RT extract (5, 10 and 25 µg/mL) from the egg stage. Then, human Aβ expression was induced by a temperature upshift that makes worms paralyze over time. The time to develop paralysis was analyzed using survival curves (Figure 4).

The time for 50% nematodes to become paralyzed (PT_50_) showed a significant increase (*p* < 0.0001) in the groups treated with RT extract compared to the untreated groups. However, between the different groups treated with RT extract, no significant differences were found in PT_50_ at 43 h. According to HR (hazard ratio) values obtained by log-rank analysis (0.76; 0.72 and 0.78), the RT extract significantly reduced the risk of paralysis by 23%, 28% and 21% in worms treated with 5, 10 and 25 µg/mL RT extract concentration, respectively.

### 3.4. Bioassays Regarding CNS Enzymes

**Figure 5:**
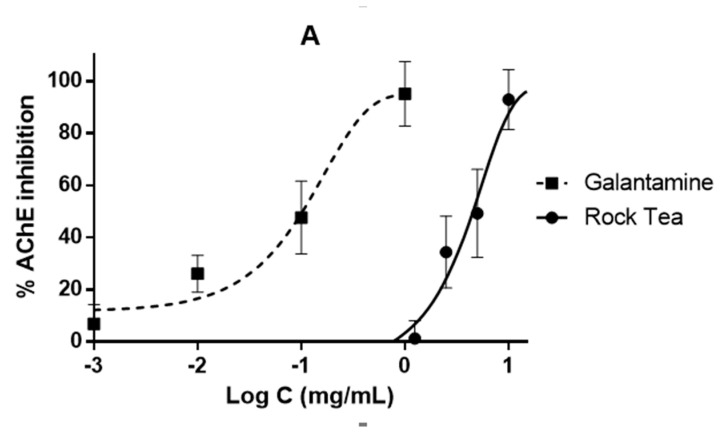

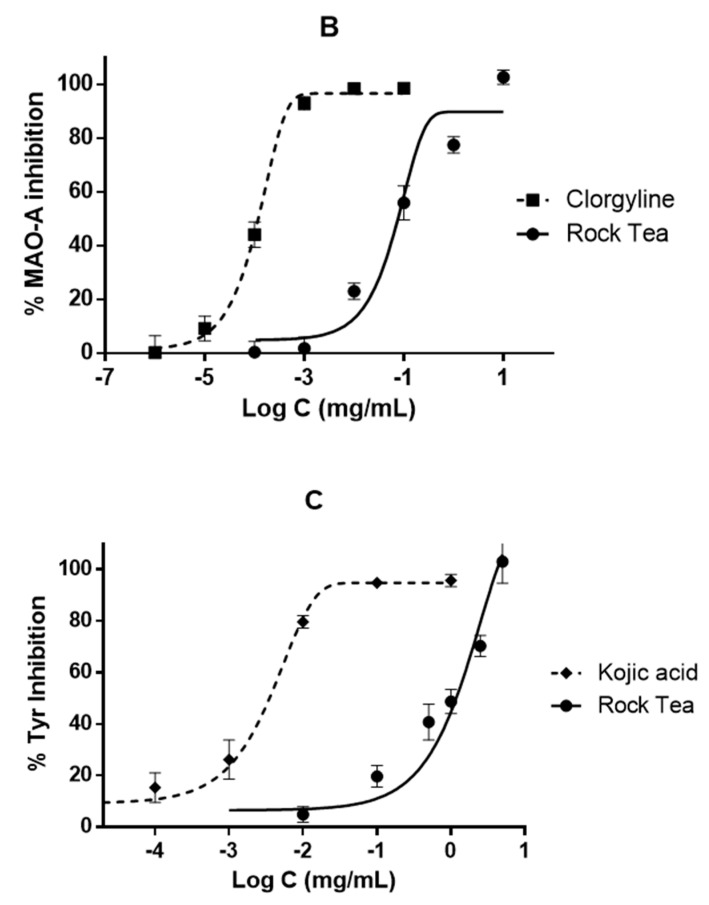
Neuroprotective effect of RT extract on nervous system enzymes; (**A**) Acetylcholinesterase (AChE) inhibition, (**B**) Monoamine oxidase A (MAO-A) and (**C**) Tyrosinase (TYR) inhibition. Galantamine, clorgyline and kojic acid have been used as reference inhibitors, respectively.

The AChE, MAO-A and TYR enzymes are involved in processes that regulate neurotransmission in the CNS. RT extract was able to inhibit all the enzymes at high doses, although the dose-response curve was shifted to the right with respect to the reference substances for each enzyme (Figure 5). AChE inhibition of RT extract is achieved at high doses, with an IC_50_ of 4.5 mg/mL for RT (Figure 5B). The galantamine IC_50_ for AChE inhibition was 0.1 mg/mL. RT extract also revealed the potential to achieve a total inhibition of MAO-A (Figure 5B). RT extract IC_50_ was 76.34 µg/mL, a little far from that of the reference inhibitor, clorgyline, which was 0.12 µg/mL. Finally, RT extract also showed the inhibitory potential of TYR in a dose-dependent manner (Figure 5C). The extract reached 100% enzyme inhibition at a high dose, 5 mg/mL, and the IC_50_ of the assay were 1.05 and 0.004 mg/mL for RT extract and kojic acid, respectively.

## 4. Discussion

This study shows, for the first time, the neuroprotective effect of *Jasonia glutinosa* extract using different types of *in vitro* and *in vivo* bioassays. The results obtained demonstrate that RT extract prevented oxidative stress, increased the lifespan and delayed paralysis of the transgenic amyloid *C. elegans*. In addition, the extract inhibited enzymes of the nervous system such as acetylcholinesterase, monoamine oxidase A and tyrosinase.

Various studies have associated reactive oxygen species with the pathogenesis of Alzheimer’s disease (AD) by showing that reactive oxygen species promote the formation and accumulation of the β-amyloid peptide and hyperphosphorylation of Tau. On the other hand, oxidative stress is associated with aging. Many theories try to explain the connection, but it is complicated due to its multifactorial etiology. The so-called “Oxi-Inflamm-Aging” theory explains the events that occur during aging and the appearance of diseases related to it; during cellular aging, senescent cells produce pro-inflammatory cytokines that produce chronic systemic inflammation (“inflamm-aging”), leading to an increase in free radicals. Similarly, oxidative stress produces states of inflammation due to the impaired immune system, creating a vicious circle between oxidative stress, inflammation and aging. This process has been implicated in multiple disease states, such as cardiovascular disease, cancer, neurodegenerative diseases, diabetes or respiratory diseases [24,25]. Oxidative stress, β-amyloid peptide accumulation the lifespan have been correlated in the *C. elegans* model [26,27], making it an excellent model to study the bioactive properties of substances with neuroprotective potential.

Different RT extracts have shown anti-inflammatory effects [4,10] and a high capacity to eliminate superoxide and DPPH radicals [4,5,9]. Furthermore, one *in vivo* study showed these antioxidant properties by decreasing HSP70 levels, increasing peroxidase activity and upregulating the Nrf-2 transcription factor, producing an increase in the expression of antioxidant enzymes such as catalase and superoxide dismutase in fish [10]. These properties, which could be explained by its rich composition in phenolic acids, flavonoids and pigments, make RT an ideal candidate as a neuroprotective natural agent.

Thus, the RT extract has demonstrated antioxidant properties and lifespan extension in *C. elegans* in a similar manner to quercetin or kaempferol, two flavonoids present in the extract. The phytochemical analysis of the ethanolic extract showed a composition rich in phenolic compounds and pigments [4]. Of the phenolic compounds identified, the most representative were the hydroxycinnamic acids derived from caffeoylquinic acid, represented mainly by 3,4-di-O, 3,5-di-O (40.95 mg/g of dry extract), 1,5-di-O (24.73 mg/g of dry extract) and 4,5-di-O caffeoylquinic acids (23.14 mg/g of dry extract). Of the flavonoids, the most representative compound was quercetin-3-O-galactoside (15.16 mg/g of dry extract). In addition, pigments in the extract were determined for the first time, highlighting the presence of carotenoids and chlorophylls, lutein being the most representative, with content greater than 55% of the total.

The treatment of *C. elegans* with quercetin or its methyl derivates, isorhamnetin and tamarixetin (200 µM), increased worm survival rate under exposure to juglone (150 µM, 24 h exposure). Quercetin showed a greater capacity than methyl derivatives in decreasing oxidative proteins compared to untreated worms. Isorhamnetine showed greater protection (16%) compared to quercetin and tamarixetine (11%), prolonging the mean lifespan of the worms compared to the control worms [13]. These results were similar to those obtained by Kampkötter and colleagues (2008), who demonstrated that quercetin (100 µM) enhanced the percentage of survival against oxidative stress (juglone 150 µM, 72 h) by 19% and the mean lifespan by 15% of worms compared to the untreated worms [28]. Furthermore, worms pre-treated with kaempferol showed diminished oxidative stress and less accumulation of reactive oxygen species [29]. Kaempferol and quercetin showed scavenging capacity in mitochondrial reactive oxygen species levels, without affecting lifespan in *mev-1* mutant worms, a mutation that increases sensitivity to oxidative stress and reduces lifespan [30]. The effect on lifespan may be explained by the antioxidant effect of these compounds and/or by other complementary mechanisms, independent of antioxidant properties, such as stress-sensitive signaling pathways.

One of the main key regulatory pathways in *C. elegans* lifespan and the response to oxidative stress is the insulin/insulin-like growth factor (IGF-1) signaling (IIS) pathway, as well as the activation of different transcription factors such as DAF-16/FoxO, HSF-1 and SKN-1/Nrf-2 that regulate stress-related genes as antioxidant enzymes. Several studies have shown that quercetin and kaempferol produced a translocation of DAF-16 from the cytosol to the nucleus in transgenic *C. elegans*, increasing the expression of defense proteins [28,29,30], the role of these DAF-16 target genes in longevity is unclear. Different studies have shown that transcription of this factor may not be responsible for the effect of flavonoids on worm lifespan, since quercetin-treated *daf-16* deletion mutants increased survival in comparison with the control [30,31,32]. It has recently been shown that resistance to oxidative stress by quercetin in worms would implicate genes involved in the IIS pathway such as *age-1*, *akt-1*, *akt-2*, *daf-18*, *sgk-1*, *daf-2* and *skn-1*, independent of transcription factors such as DAF-16 and HSF-1.

Other important compounds in RT extract are caffeoylquinic acids (CQAs), which are emerging as an interesting group of bioactive compounds with high potential as neuroprotective agents. These compounds have been shown to possess strong antioxidant and neuroprotective properties *in vitro* and *in vivo*. CQAs have also been shown to improve cognitive impairment in several rodent models of AD [33]. Recently, Chen et al. [34] showed that 1,5-O-dicaffeoyl-3-O-(4-malic acid methylester)–quinic acid (MQA), a derivative of caffeoylquinic acid, can protect against ischemic brain injury in rats. This neuroprotective effect may involve the inhibition of cell apoptosis due to p38 activation, the increase of the Bcl-2/Bax ratio and the modulation of NFk-B1 and caspase-3 expression. Furthermore, the observed decrease in lipid peroxidation suggests that the antioxidant activity of MQA could also contribute to the protective effect against cerebral ischemia. The association between antioxidant effects and neuroprotection has been previously demonstrated for other CQAs. Thus 3-O-caffeoylquinic acid and caffeic acid treatment improved memory in the mouse model of Aβ accumulation by the inhibition of oxidative stress, inflammation and apoptosis through the p38 mitogen-activated protein kinase (MAPK) signaling pathway [35,36]. Similarly, Chu et al. [37] observed that a flavone-rich extract of *Tetrastigma hemsleyanum* whose major components were 3-caffeoylquinic acid, 5-caffeoylquinic acid and quercetin-3-O-rutinoside, and kaempferol-3-O-rutinoside could attenuate glutamate-induced toxicity in PC12 cells and *C. elegans*. This extract lessened genotoxicity, relieved oxidative stress and recovered mitochondrial functions in PC12 cells via the MAPK pathway by suppressing the over-phosphorylation of ERK and p38. Furthermore, treatment suppressed O_2_^-^ generation, reduced GSH depletion and partially restored the normal motility lost after the glutamic acid exposition in *C. elegans*. Therefore, antioxidant properties could explain the positive effect of *J. glutinosa* extract in increasing the lifespan in N2 and transgenic *C. elegans* (CL4176 strain).

As mentioned above, oxidative stress and the malfunction of specific enzymes related to neurotransmitter effects, as AChE, are involved in the development of the pathogenesis of AD. The accumulation of Aβ peptide caused by free radicals would result in neuron dysfunction and death. Different works have shown that phenolic compounds present neuroprotective activity, decreasing the deleterious effects of oxidative stress, increasing ACh availability, reducing the anti-inflammatory effect and interacting directly with Aβ peptides inhibit their aggregation and oligomerization [15,23,38,39]. The reduction of the IIS pathway and expression of genes as DAF-16, HSF-1, HSP-16 and Nrf2/SKN-1 have also been related to a neuroprotective effect by decreasing the expression of Aβ peptides and improved the paralysis and lifespan in a *C. elegans* model of AD [40]. These results show that RT extract decreases the risk of paralysis in transgenic strain CL4176, and its neuroprotective effect can be explained by the synergic antioxidant and neuroprotective activities of phenolic acids and flavonoids.

On the other hand, *C. elegans* present numerous neurotransmitters, some of which are implicated in different pathologies of the nervous system such as ACh, dopamine, GABA or glutamate [41]. RT extract has demonstrated the ability to inhibit the AChE, MAO and TYR enzymes, which could explain its traditional uses as a stimulant, memory enhancer or antidepressant, reinforcing its neuroprotective role. Others isolated extracts of plants and flavonoids have demonstrated the capacity to inhibit CNS enzymes [15,18,23,42,43] and decrease the toxicity by Aβ accumulation [15,23,38,44]. Due to these mechanisms, many natural products have neurotherapeutic potential for the prevention and treatment of different CNS disorders.

## 5. Conclusions

*Jasonia glutinosa* is a medicinal plant with a wide range of traditional uses. The neurotherapeutic potential of a polyphenolic extract has here been demonstrated in different models due to its protective activity against oxidative stress and the capacity to inhibit β-amyloid aggregation in *C. elegans*. This herbal extract has also shown the inhibitory capacity of nervous system enzymes, which might explain, from an *in vitro* perspective, possible mechanisms of its use as a stimulant and antidepressant. All in all, this plant species as well as its extracts and bioactive compounds are worthy of further investigation for the prevention of diseases associated with cellular aging and oxidative stress.

## Data Availability

The data presented in this study are available in this article.

## List of Abbreviations

AChE: acetylcholinesterase, AD: Alzheimer’s disease, Aβ: amyloid-β, CQAs: caffeoylquinic acids, NGM: nematode growth media, MAO-A: monoamine oxidase A, MQA: 1,5-O-dicaffeoyl-3-O-(4-malic acid methylester)–quinic acid, RT: rock tea, TYR: tyrosinase, % SR: survival rate %.
